# Undergraduate Medical Teachers’ Perceptions Toward Video-Based Feedback in Teaching Communication Skills: A Qualitative Study Protocol

**DOI:** 10.7759/cureus.95023

**Published:** 2025-10-21

**Authors:** Wen Wei Lim, Cai Paris Li Min

**Affiliations:** 1 Medicine, Hull University Teaching Hospitals Trust, Hull, GBR; 2 Medicine, International Medical University, Kuala Lumpur, MYS; 3 Vascular Surgery, Hull University Teaching Hospitals Trust, Hull, GBR

**Keywords:** communication skills, medical education, qualitative protocol, thematic analysis, video‑based feedback

## Abstract

Background and objective

While effective communication is a core competency in medical education, undergraduate instructors often lack structured methods to deliver feedback on this skill. This study protocol aims to explore undergraduate medical teachers’ perceptions regarding using video-based feedback to enhance communication skills teaching.

Methods

We plan to conduct semi-structured interviews with 9-17 undergraduate teachers from a private medical university, lasting 30-45 minutes each, which will be audio-recorded, transcribed verbatim, and anonymized. Thematic analysis will be conducted by following Braun and Clarke’s six-phase method, with coding performed independently by two researchers and discrepancies resolved through discussion.

Results

We anticipate identifying themes related to perceived benefits (e.g., improved self-reflection), potential challenges (e.g., time constraints, technological barriers), and actionable suggestions for implementation.

Conclusions

We believe the findings will inform the design and integration of video-based feedback tools to strengthen communication skills teaching in undergraduate medical curricula.

## Introduction

Video-based feedback (VBF) is a teaching approach that involves recording a student’s performance during a patient encounter, which is later reviewed collaboratively by the student, peers, and instructor in a face-to-face setting [1]. The teacher may pause the video at intervals to encourage the student’s self-assessment, invite peer feedback, or offer their own observations. Van der Kleij [2] suggested that the method of feedback delivery by teachers has minimal effect on how students perceive it. Teachers generally believe they provide useful feedback, yet many students do not find this feedback helpful, reporting that feedback is often vague and difficult to apply [2,3]. This highlights a disconnect between how feedback is intended and how it is received and a discrepancy between teachers' and students' perceptions of feedback [2,3]. The disconnect between teachers’ intentions in feedback and students' perceptions of its value can hinder learning [2].

There is scarce research exploring the perceptions of VBF [4]. Students generally view VBF as a valuable learning tool [1,5]. Although VBF is widely used in medical training, published studies predominantly examine learner experiences and outcomes rather than educators’ perspectives on feasibility, value, and implementation barriers in communication-skills teaching ​[1,6,7]. More research is required to uncover teachers’ perceptions of VBF [8]. Exploring teachers’ perspectives could provide insights into why this discrepancy exists, enabling adjustments in how feedback is delivered. Furthermore, publishing this protocol enhances methodological transparency and reproducibility for a qualitative thematic analysis, aligning with best-practice guidance in medical education research.

Experiential learning is defined as a cyclical process in which knowledge is constructed through the transformation of experience, involving four stages: Concrete Experience, Reflective Observation, Abstract Conceptualisation, and Active Experimentation [9]. In the context of VBF, video-recorded consultations may improve learning by giving students opportunities to assess their interactions and reactions in clinical settings. It is the underpinning medical education theory, as Kolb's learning cycle directly aligns with the processes enabled by VBF. Reviewing these videos can promote self-awareness and critical evaluation of communication skills, and help students develop concepts of successful communication that they can apply in future interactions.

This theory guides the formulation of the research question and the interview schedule by focusing on teachers’ experiences with VBF, what teachers think the challenges associated with using VBF as a learning tool are, and how to overcome those challenges. The extent to which teachers think VBF supports experiential learning can also be explored. Obtaining these insights will help improve the integration of VBF into communication skills lessons as part of undergraduate medical education and create more efficient learning environments. Furthermore, teachers’ insights can help identify areas where extra resources are required to maximise the effectiveness of VBF as a teaching tool. For example, if teachers express concerns about managing the technological parts of VBF, training might be implemented to boost their competency.

## Materials and methods

This research aims to understand how medical teachers perceive VBF and to provide recommendations to improve the use of VBF in the feedback process for students at a UK medical university. The research seeks to understand the benefits of incorporating VBF into teaching communication skills and other clinical skills, and to understand the challenges faced by teachers when using VBF in teaching communication skills.

This study will be conducted as a low ethical risk primary investigation using qualitative descriptive methodology [10]. Qualitative description is particularly suitable for exploring experiences and gathering insights from participants about phenomena that are not well understood [10]. This approach is appropriate given the subjective nature of the research topic and the anticipated diversity in participants’ experiences with VBF [11].

Alternative qualitative methodologies were considered but found to be less suitable in this context. Constructivist Grounded Theory, which prioritises theory generation from data independent of existing frameworks, was deemed inappropriate because the current research aims to explore perceptions rather than develop new theoretical constructs [12]. Similarly, Phenomenology, which focuses on deeply interpreting lived experiences and the meanings assigned to them, was considered less aligned with the study's objective of exploring general patterns of perception around the use of VBF, rather than conducting detailed personal meaning analysis [13].

Study population and recruitment

Purposive sampling will be used to identify and recruit participants who are likely to offer rich, relevant insights into the topic under investigation [14]. This method is chosen to optimise resources and to specifically target educators involved in planning or delivering VBF within the university. All educators who have used VBF in undergraduate communication skills teaching will be invited to participate.

An administrative staff member from the university’s medical education centre will act as a gatekeeper, distributing standardised email invitations. This individual will not influence participation in any way. A uniform invitation email will be sent to all potential participants, ensuring neutral language that does not promote or discourage participation. The invitation period will span two weeks, with a reminder email issued in the second week. Participation will be entirely voluntary. If the initial response is limited, additional recruitment rounds or snowball sampling will be employed to ensure adequate representation and data saturation. Exclusion criteria include individuals who do not possess the necessary technological means (e.g., a computer and a webcam) required to participate in virtual interviews.

The number of interviews will be guided by data saturation, defined as the point at which no new themes emerge during analysis [15]. Sample adequacy will be guided by saturation, monitored via code saturation, which occurs when no additional codes are identified; and meaning saturation, which occurs when no further elaboration of existing themes is found [16]. This range is appropriate given the study’s specific research questions and targeted scope [16]. We will review saturation iteratively after the ninth interview and then after every two interviews, documenting new codes and theme elaborations. Prior work indicates that for focused questions with relatively homogenous participants, 12 interviews often capture most codes and 9-17 interviews are typically sufficient to reach meaning saturation ​[16,17,18].

The semi-structured interview guide was developed iteratively from the VBF literature and our experiential-learning framing, based on practical advice for writing interview schedules and probes that elicit rich data without leading questions ​[19,20]. We will revise the protocol to include piloting the interview schedule with two eligible educators who are not included in the main sample to refine wording, order, flow, and probe utility ​[19]. Table 1 below depicts the interview schedule that will be followed.

**Table 1:** Interview questions

Semi-structured interview schedule
Can you tell me about your experience with using video-based feedback (VBF) in your teaching? Can you share an example of a time when you found VBF particularly effective or ineffective? How valuable do you find the content of the video feedback that you provide?
How do you feel about using VBF in teaching communication skills? What aspects do you find most effective? Are there aspects you find less effective or difficult to manage?
Can you describe how students typically respond to receiving VBF? In your experience, how do students respond to VBF? What aspects of VBF do you think are most effective in facilitating this understanding? Have you noticed any differences between how you and your students perceive the value of VBF?
What do you think are the main benefits of using VBF in your teaching? Conversely, have you experienced any drawbacks or concerns when using VBF?
What recommendations would you make to improve the use of VBF in teaching communication skills? If you could make changes to how VBF is implemented, what would they be? Are there any resources, training, or tools that could enhance your ability to use VBF effectively?
Have you encountered any challenges or limitations with VBF? How do you address these challenges, and what solutions do you think might improve the process? If you could make changes to how VBF is implemented, what would they be? Do you think these challenges outweigh or balance the benefits?

Data collection strategy

One-to-one semi-structured interviews will be conducted to explore participants’ experiences and perspectives in depth [17]. Focus groups will not be used due to scheduling challenges and the risk of groupthink, where group consensus may obscure individual perspectives [21]. An interview schedule will guide each session and contain pre-developed open-ended questions to ensure consistency across interviews while allowing space for elaboration [21]. This format enables a methodical yet flexible approach to data gathering that maintains focus while encouraging natural conversation [21]. The questions will be carefully sequenced to facilitate flow and reflect the research objectives [19].

Unstructured interviews will not be used, as their broad nature complicates data analysis and can be difficult to manage by both researchers [20,14]. All interviews will be conducted using the Zoom platform, recorded via an encrypted computer, and transcribed verbatim. Transcripts will be anonymized and stored in a password-protected folder on a secure computer.

Data analysis strategy

Data will be analyzed using Thematic Analysis, a method for identifying, analyzing, and reporting recurring patterns (themes) within qualitative data [17]. This method is suitable for capturing a wide range of perspectives and making sense of collective experiences within the dataset [18]. 

Qualitative data analysis software (e.g., NVivo) will be used to support systematic coding and reduce the risk of human error during analysis [22]. To enhance rigor, we will follow Lincoln and Guba’s framework of trustworthiness, which addresses credibility, transferability, dependability, and confirmability [23]. In terms of credibility, it will be promoted through prolonged data engagement, as the researcher will conduct all interviews and personally transcribe them to ensure familiarity [17]. This will be supported by peer-debriefing sessions to cross-check coding and interpretations [24]. We will return a plain language summary of preliminary themes to participants and invite them to provide comments. Transferability will be supported by providing a thick description of participant characteristics, institutional context, and verbatim extracts. This allows readers to determine applicability to their own settings. Dependability will be promoted by maintaining a transparent audit trail that includes a versioned codebook and reflexive journaling to record any changes in assumptions [24]. Confirmability will be ensured by linking all analytic claims to data extracts and engaging in reflexive practice to reduce researcher bias.

The analysis will follow the six-phase framework proposed by Braun and Clarke [17]: familiarization with data refers to where the researcher will personally transcribe the audio recordings, read through transcripts, and record initial thoughts. The first author will transcribe all interviews to support immersion, and NVivo will be used to organize coding. A second researcher will independently code a subset of transcripts, with differences resolved collaboratively [24]. Peer-debrief sessions will be held throughout to review coding and theme development, providing triangulation and an audit trail.

All interviews will be transcribed verbatim by the first author using digital audio files and checked against the recordings to ensure accuracy. We will use the software, NVivo Transcription, to assist in generating draft transcripts, and manual review and correction will be undertaken to maintain fidelity to participants’ words. During the familiarization stage, the first author will also keep a reflexive journal while transcribing and reviewing the interview transcripts. The notes will log observations and tentative ideas for codes that arise during this stage. The journal entries will be dated and stored as part of the study’s audit trail to provide a record of how interpretations developed over time. By revisiting these reflections throughout the analysis, the team can ensure that coding decisions remain transparent and grounded in the data, while also acknowledging the influence of the researcher’s perspective [24].

Step two will involve systematic coding, where data will be coded using software to identify key features across transcripts. Thirdly, we will engage in theme generation to group related codes to form preliminary themes. The fourth step will entail theme review, where themes will be checked against coded data extracts and the full dataset to ensure relevance and coherence. Step five will involve theme refinement and naming, where themes will be refined for clarity and named to reflect their essence. Step 6 will be report production, where extracts will be selected to illustrate key findings and contribute to an analytic narrative. A table illustrating the steps of Thematic Analysis is outlined in Table 2 below.

**Table 2:** Six-phase thematic analysis process (adapted from Braun and Clarke, 2006)

Phase (Braun and Clarke)	What we will do	Output kept for audit
1. Familiarisation	Primary researcher transcribes/checks audio; both researchers read transcripts and jot initial impressions.	Read-through memos, transcript notes
2. Generating codes	Inductive, semantic-first coding in NVivo; short, action-oriented labels (e.g., “time cost of set-up”).	Initial code list, code notes
3. Constructing themes	Cluster related codes; sketch candidate subthemes/themes; map relations.	Theme maps, theme memos
4. Reviewing themes	Check themes against extracts and whole dataset; seek negative or deviant cases; refine boundaries.	Theme review log, anchor extracts
5. Defining and naming	Write concise names; definitions with inclusion and exclusion rules for each theme.	Theme definitions sheet
6. Producing report	Select vivid, varied quotes. Link themes to research questions and implications.	Quote bank, report excerpts for me to copy and paste

Ethical considerations

To ensure confidentiality, participants will have access to an information leaflet to make an informed decision to participate in the study. Participants’ personal identifying characteristics will be removed to de-identify them. Each participant’s audio recording will be labelled with a code number and a conversion file stored in an encrypted, secure computer. All data will be encrypted using encryption software to convert readable data into an encoded format that can only be accessed by the primary researcher. The recordings will be deleted within a week after transcription for data protection purposes. 

To ensure data trustworthiness, we will pseudonymize transcripts, separate the ID-key, and store encrypted files on an encrypted computer ​[25]. We will use a semi-structured guide with neutral probes to minimise leading and preserve participants’ language, and we will document any refinements from piloting in our decision trail ​[19,20]. Furthermore, a codebook will be used to ensure every alteration is traceable and provide a robust audit trail. This transparent chain of evidence supports dependability and confirmability in reflexive thematic analysis ​[15].

To ensure voluntary participation, there will be no coercion or materialistic enticement during the recruitment process. All participants have the right to opt out of the research by sending the primary researcher an email, and their collected data will be discarded afterward. This study will undergo the ethical approval process of the institution from where the participants will be recruited.

Reflexivity statement

Reflexivity is a process where researchers continuously evaluate how their subjectivity could affect their research [26]. I experienced video-based feedback in a communication skills class and noticed that the activity was well received; thus, I would like to understand how teachers perceive it.

I am a resident doctor who will be interviewing participants who are at a similar or higher ranking than me, which may be influenced by power dynamics. Power dynamics refer to the imbalance that arises when researchers hold positions of authority over participants, potentially shaping the nature and quality of the data collected [26]. However, interviewees can actively respond to this power imbalance, employing strategies to influence the interview outcomes [27]. For example, the interviewee holding a position of authority may decide to terminate the interview prematurely ​[27]. Strategies to mitigate this include setting an agenda on the topics to be discussed so that I can control the direction of the interview ​[27]​.

This research topic stemmed from my belief that video-based feedback is an excellent method of feedback provision. This belief may be challenged if participants provide negative responses during interviews. It is my duty to manage information shared by participants, and consider their responses both beforehand and in the moment ​[28]. Therefore, I need to be aware of my emotions when this happens by maintaining a reflexive journal. This exercise helps prepare me for interactions with participants by helping me define my motives for the research, life experiences, and reflect on how my experiences may affect my understanding of participants’ responses ​[26].

## Results

Since this is a protocol, data collection is pending. However, we anticipate that emerging themes will include perceived benefits such as improved teaching self-awareness, enhanced quality of feedback to students, and increased reflective teaching. Potential challenges may include concerns about time investment, technical infrastructure, discomfort with self-observation, and concerns about peer judgment. Contextual factors expected to emerge include the importance of institutional support, allocated protected time, training in video technology, and clear guidelines for data security and confidentiality.

## Discussion

This study protocol outlines a qualitative descriptive design aimed at exploring undergraduate medical teachers’ perceptions of video-based feedback (VBF) as a tool for teaching communication skills. The use of VBF is increasingly common in undergraduate medical education, offering opportunities for reflection, self-assessment, and formative feedback through the review of recorded clinical encounters. Despite some evidence indicating that students perceive VBF positively due to its ability to highlight nonverbal communication and facilitate self-awareness [1], there remains a notable gap in understanding how educators perceive this modality, particularly in relation to its practicality, educational value, and alignment with experiential learning theories such as Kolb’s learning cycle [8].

Existing literature highlights a mismatch between how feedback is delivered and how it is received by students. Teachers often believe they provide meaningful feedback, yet students may find it vague or difficult to apply [7]. This disconnect suggests that the method of feedback delivery, including the use of VBF, warrants deeper exploration from the teacher’s perspective [7]. While studies such as those by Dohms et al. [1] demonstrate student enthusiasm for VBF, there is a lack of robust qualitative research capturing educators’ views, particularly regarding implementation barriers, comfort levels, and perceived impact on student engagement.

This protocol employs semi-structured interviews to elicit detailed insights from educators directly involved in VBF delivery. The use of Braun and Clarke’s thematic analysis framework [17] will allow for systematic exploration of patterns and themes within the data, providing a nuanced understanding of the educational affordances and limitations of VBF in this setting. The interview schedule is informed by existing gaps in the literature and aligned with experiential learning theory to explore how VBF supports reflective observation and conceptualisation phases of Kolb’s cycle [8].

The design of this study is grounded in several notable strengths. Firstly, qualitative description is well-suited to exploratory research involving varied and subjective perspectives [10,11,29]. Secondly, purposive sampling will ensure that participants with direct experience in VBF are included, optimising the depth and relevance of the data [14]. Furthermore, efforts to minimise bias, such as the use of a standardised email template, voluntary participation, and ethical safeguards, enhance the credibility and trustworthiness of the findings.

Nonetheless, certain potential limitations must be acknowledged. The study will be confined to a single institution, which may limit generalisability. There is also a possibility of selection bias, as educators with strong opinions - either positive or negative - may be more inclined to participate. Additionally, power dynamics during interviews may influence how openly participants share their views, particularly if the interviewer is known to them or perceived to be junior in hierarchy** **[26].Strategies such as pre-interview rapport-building and reflexive journaling will be employed to mitigate these effects [26].

The findings from this study are expected to inform practical recommendations for improving the delivery of VBF in undergraduate medical curricula. By highlighting perceived barriers and facilitators, the results may guide future training programs for faculty, the development of institution-specific guidelines, and the integration of VBF in a way that aligns with both educator capabilities and student learning needs. Moreover, these insights may support broader institutional adoption of VBF as a reflective learning tool, especially if supported by appropriate technological infrastructure and faculty development initiatives.

Limitations

Participants will be recruited through voluntary response to an institutional invitation, which may lead to selection bias, as educators more confident or experienced with VBF may have been more inclined to participate. Consequently, findings may over-represent positive perceptions. Nonetheless, purposive sampling was appropriate for the exploratory nature of this study, and the insights generated provide valuable context for future work incorporating a broader range of participants.

## Conclusions

This protocol outlines a qualitative study designed to explore undergraduate medical teachers’ perceptions of VBF in teaching communication skills. By capturing educators’ experiences, perceived benefits, and challenges, the study aims to inform the effective integration of VBF into medical education. The findings are expected to provide practical insights that support faculty development, enhance feedback delivery, and strengthen reflective learning within the curriculum.
